# Comparison of domains of self-reported physical activity between Kenyan adult urban-slum dwellers and national estimates

**DOI:** 10.1080/16549716.2017.1342350

**Published:** 2017-07-18

**Authors:** Hilde E. Groot, Stella K. Muthuri

**Affiliations:** ^a^ Departments of Population Dynamics and Reproductive Health, African Population and Health Research Center, Nairobi, Kenya; ^b^ University of Groningen, University Medical Center Groningen, Groningen, The Netherlands

**Keywords:** Self-report, physical activity, non-communicable diseases, national, slum, Kenya

## Abstract

**Background**: Non-communicable diseases (NCDs) – largely the result of modifiable behavioral risks such as physical inactivity that gradually develop into physiological risks – are a main cause of morbidity and mortality worldwide. In Kenya, a nationally representative STEPwise survey of risk factors for NCDs established that 10.8% of Kenyans accumulated low levels of total physical activity.

**Objectives**: The goal of our analyses was to compare domains of self-reported physical activity in two Nairobi slums to national estimates.

**Methods**: Levels and time of self-reported activity in three domains (work, transport, and recreation), collected as part of a SCALE-UP study conducted in Korogocho and Viwandani slums in Nairobi, were compared to STEPwise findings.

**Results**: The samples included a total of 10,128 participants (5,628 slum, 4,500 national). Only 7.1% and 4.0% of slum dwellers reported low levels of work and transport physical activity, respectively, but 95.9% reported low levels of recreation-related activity. Slum residents reported higher mean daily minutes of total activity than the national estimate (499 minutes versus 291 minutes), however, both samples spent similar proportions of total activity on work (79.0% slum, 78.3% national), transport (20.4% slum, 18.1% national), and recreation (0.6% slum, 3.6% national) activities.

**Conclusions**: While the total amount of time spent in different domains of self-reported activity differs between urban slum residents and the national Kenyan population, proportions of time in each of the three domains are similar. It is important that such differences or similarities be considered when addressing NCD risk factors in these populations.

## Background

Non-communicable diseases (NCDs) resulted in a staggering 38 million of the total 56 million deaths that occurred in 2012, with three of every four NCD related deaths recorded in low- and middle-income countries (LMICs). Four types of NCDs are major contributors to these deaths, namely cardiovascular diseases (17.5 million or 46.2% of NCD deaths), cancers (8.2 million or 21.7% of NCD deaths), respiratory diseases, such as asthma and chronic obstructive pulmonary disease (4 million or 10.7% of NCD deaths), and diabetes (1.5 million or 4% of NCD deaths). Regrettably, NCD related deaths are expected to continue rising, with cardiovascular disease (CVD) mortality – the leading cause of mortality worldwide – expected to increase from 17.5 million in 2012 to 22.2 million in 2030. Over 80% of CVD deaths occurred in LMICs and this rate is projected to increase in the upcoming years [1].

The increasing burden of NCDs in LMICs can largely be explained by epidemiological transition theory, which provides a framework for understanding changes in disease patterns as a result of socio-economic and behavioral changes. Urbanization and increased socioeconomic standing, or a move from more traditional lifestyles, has resulted in increased access to motorized transport and less active transportation, more sedentarism, through increased numbers of desk jobs as opposed to trades requiring physical effort, and increased access to and intake of higher caloric content and processed foods and consequently less vegetable and fruit consumption [2,3].

Even more concerning for many LMICs, such as those in sub-Saharan Africa, is that while they start to tackle this new burden of NCDs, they are also simultaneously dealing with infectious diseases, a phenomenon known as a ‘double burden of disease’ [4]; it is clear that prevention of NCDs is urgently needed.

The majority of NCD related deaths are the result of four risk behaviors, namely unhealthy diets, physical inactivity, tobacco use, and the harmful use of alcohol, which may result in four main metabolic risks, namely high blood pressure or hypertension, high blood glucose, high cholesterol, and overweight/obesity. Fortunately, the four behavioral risks are modifiable, meaning intervention can reverse the detrimental health effects. The World Health Organization (WHO) suggests various ways to achieve this, including limiting tobacco and alcohol use, reducing sugar, salt, and fat intake, ensuring sufficient consumption of fruit and vegetables, and attaining adequate physical activity levels [5]. For health benefits, it is recommended that adults aged 18–64 years accumulate at least 150 minutes of moderate-intensity or at least 75 minutes of vigorous-intensity physical activity throughout the week, or an equivalent combination of both [5].

In 2015, results from the Kenya STEPwise survey – the first nationally representative survey aimed at collecting comprehensive information on risk factors for NCDs, injuries, and oral health in adults aged 18–69 years in the country – were published. The aim of the survey was to establish an NCD surveillance platform to collect indicators on determinants of NCD for policy and planning purposes [6]. The findings showed that 10.8% of Kenyans (9.0% in rural areas and 13.8% in urban areas) reported low levels (rather than moderate or high levels) of physical activity considering work-, transport-, and recreation-related activities. It was further established that 78.3% of total physical activity reported was work-related, 18.1% transport-related, and 3.6% recreation-related [6].

The STEPwise national survey made rural-urban distinctions in levels of activity, however, it is possible that levels of physical activity could differ between urban slum residents, other areas in Nairobi and the national estimates. Recently, it has become clearer that slum health is incomparable to the overall health of a certain country. In Kenya, the health outcomes of urban residents have been found to be worse than their non-slum counterparts, and comparable or worse than that of rural dwellers [7]. As the slum population increases in coming years, it is important that focus is placed specifically on this growing population, such that strategies to improve their health and wellbeing are context specific [8–10]. Hence, the goal of the analyses herein was to compare domains of self-reported physical activity (including work, transport, and recreational activities), in two Nairobi slums to the national prevalence.

## Methods

### Study design and population

Residents of two slums, Korogocho and Viwandani in Nairobi, Kenya, participated in a prospective evaluation study (SCALE-UP), conducted by the African Population and Health Research Center (APHRC) previously [10]. The aim of the SCALE-UP study was to evaluate the impact of a community-based CVD prevention program on blood pressure and other CVD risk factors among urban slum dwellers. Respondents were adults aged 35 years or older, who were residents in either of the two slums with the ability and willingness to give informed consent to participate in the study. Exclusion criteria included pregnancy, a history of CVD (myocardial infarction, stroke, heart failure, or angina pectoris), psychiatric illness, and inability to provide informed consent. For the nationally representative STEPwise survey, demographic and socio-economic data were collected during household visits by trained field workers. Furthermore, anthropometric measures, including hip, waist, height, weight, and blood pressure, were collected as the study baseline in August 2012. Participants were adults aged 18–69 years and were required to give informed consent prior to participation. Similarly, the SCALE-UP study entailed household visits and face to face interviews with participants aged 35 years and older; field work was carried out between April and June 2015 by a well trained team. Every visit started with gathering key information on NCD risk factors via a questionnaire, then proceeded to collection of direct physical measurements and blood samples for biochemical analysis [6]. Questionnaires included detailed questions on lifestyle regarding CVD risk factors such as diet, physical activity, and tobacco and alcohol use. As shown in Table 1, the physical activity related questions were similar to those used in the STEPwise survey.

**Table 1.** Questions on various domains of physical activity.

Domain of Activity	Questions
Work-related	Does your work involve vigorous-intensity activity that causes large increases in breathing or heart rate like carrying or lifting heavy loads, digging or construction work for at least 10 minutes continuously? In a typical week, on how many days do you do vigorous-intensity activities as part of your work? How much time do you spend doing vigorous-intensity activities at work on a typical day? Does your work involve moderate-intensity activity that causes small increases in breathing or heart rate such as brisk walking, carrying light loads, for at least 10 minutes continuously? In a typical week, on how many days do you do moderate-intensity activities as part of your work? How much time do you spend doing moderate-intensity activities at work on a typical day?
Recreation related	Do you do any vigorous-intensity sports, fitness or recreational (leisure) activities that cause large increases in breathing or heart rate like running or football, for at least 10 minutes continuously? In a typical week, on how many days do you do vigorous-intensity sports, fitness or recreational (leisure) activities? How much time do you spend doing vigorous-intensity sports, fitness or recreational activities on a typical day? Do you do any moderate-intensity sports, fitness or recreational (leisure) activities that cause a small increase in breathing/heart rate like cycling or swimming for at least 10 minutes continuously? In a typical week, on how many days do you do moderate-intensity sports, fitness or recreational (leisure) activities? How much time do you spend doing moderate-intensity sports, fitness or recreational activities on a typical day?
Transport related	Do you walk or use a bicycle for at least 10 minutes continuously to get to and from places? In a typical week, on how many days do you walk or use a bicycle for at least 10 minutes continuously to get to and from places? How much time do you spend walking or cycling on a typical day?

### Statistical analysis

To allow comparability of SCALE-UP data to the STEPwise findings, discrete variables were presented as frequencies and percentages (unadjusted). Regarding the physical activity questions, ‘vigorous-intensity activities’ were considered activities that require hard physical effort and cause large increases in breathing or heart rate, 'moderate-intensity activities' are activities that require moderate physical effort and cause small increases in breathing or heart rate. ‘Low-intensity activities’ were considered as a category per exclusionem (so, no ‘vigorous-’and/or ‘moderate-intensity activities’ 6. This information was given before the participants had to answer these questions. Furthermore, examples were given each question (Table 1). Depending on the self-reported answers the participants gave, we categorized them in low, moderate, or vigorous activity. Statistically significant group differences were considered at a 2-tailed p-value of <0.05. All statistical analyses were performed with Stata version 13.0 (StataCorp).

## Results

Baseline characteristics for participants of the SCALE-UP (*n *= 5628) and STEP-UP (*n *= 4500) studies are presented in Table 2. The age distribution lay towards older ages for slum residents; for instance, the proportion of those 44 years and younger was 48.0% for slum dwellers, and 71.4% for the national sample, and the age group 45–59 years in the slum sample was double that of the national sample. Fewer participants in the slum sample (22.0%) had attained a secondary or higher level of education than the national sample (31.4%).

**Table 2.** Baseline characteristics.

	SCALE-UP Study Urban Slums (*n* = 5,627) *n (%)*	STEPwise Survey National (*n* = 4,500) *n (%)*
Sex		
Male	3,101 (55.1)	1,799 (40.0)
Age		
18–29		1,498 (33.3)
30–44	2,702(48.0)	1,713 (38.1)
45–59	2,172 (38.6)	875 (19.4)
60–69	490 (8.7)	414 (9.2)
>70	263 (4.7)	-
Educational level		
Less than primary school	979 (17.4)	1,607 (35.7)
Primary school complete	2,710 (48.2)	1,480 (32.9)
Secondary school and above	1,237 (22.0)	1,413 (31.4)
Missing	701 (12.4)	-

Overall, both male and female slum residents reported spending most of their time on work- and transport-related physical activity in a typical week, however, men spent more time on high rather than moderate level work-related activity, with the inverse found for women (Table 3). Taken together, 49.1% of respondents (40.0% men, 60.1% women) spent time in moderate level work-related activity, and 43.8% (54.3% men, 31.0% women) spent time in high level work-related physical activity. As expected, the oldest age group were least active, as shown in Table 3.

**Table 3.** Work-related activity among urban slum residents (SCALE-UP study).

Age group (years)	*n*	Low (%)	95% CI	Moderate (%)	95% CI	High (%)	95% CI
							
30–44	**2702**	3.7	2.9–4.2	48.7	46.9–50.6	47.5	45.6–49.3
45–59	**2172**	5.6	4.6–6.5	48.7	46.6–50.8	45.7	43.4–47.6
60–69	**490**	14.7	11.8–18.1	54.7	50.1–58.9	30.6	26.5–34.6
> 70	**263**	33.7	28.0–39.4	47.4	41.2–53.2	18.9	14.4–23.8
**Total (ALL)**	**5627**	**6.9**	**6.1**–**7.4**	**49.3**	**47.8**–**50.4**	**43.8**	**42.5**–**45.0**
							
30–44	**1432**	3.1	2.2–4.0	35.7	33.1–38.1	61.2	58.6–63.6
45–59	**1277**	4.4	3.1–5.4	41.7	38.8–44.2	53.9	51.1–56.6
60–69	**276**	11.8	8.3–15.9	54.5	48.4–60.2	33.7	28.4–39.5
> 70	**117**	29.4	21.5–38.0	47.9	38.9–56.9	22.7	15.6–30.7
**Total (MEN)**	**3101**	**5.5**	**4.5**–**6.1**	**40.1**	**38.4**–**41.8**	**54.4**	**52.5**–**56.0**
							
30–44	**1271**	4.2	3.1–5.2	63.6	60.9–66.2	32.2	29.5–34.6
45–59	**895**	7.4	5.7–9.2	58.9	55.6–62.1	33.7	30.6–36.8
60–69	**214**	18.8	14.0–24.5	54.8	47.9–61.2	26.4	20.7–32.5
> 70	**146**	37.3	29.5–45.1	46.9	38.6–54.7	15.8	10.7–22.6
**Total (WOMEN)**	**2526**	**8.6**	**7.4**–**9.6**	**60.3**	**58.2**–**62.0**	**31.1**	**29.2**–**32.8**

Abbreviations: CI (confidence interval). Percentages and CI’s are unadjusted.

Overall, the respondents were spending very little time in moderate and high level recreation-related physical activity, with only 4.1% of the slum residents (5.3% men, 2.8% women) spending time in moderate or high level recreational activity, as shown in Table 4.

**Table 4.** Recreation-related activity among urban slum residents (SCALE-UP study).

Age group (years)	*n*	Low (%)	95% CI	Moderate (%)	95% CI	High (%)	95% CI
							
30–44	2702	94.9	94.0–95.7	2.4	1.8–2.9	2.6	2.0–3.1
45–59	2172	95.8	94.8–96.5	2.4	1.6–2.9	1.8	1.1–2.2
60–69	490	95.9	93.3–97.0	3.2	1.7–4.8	0.9	0.2–1.9
> 70	263	96.4	93.1–97.9	3.6	1.5–6.0	0	-
**Total (ALL)**	**5627**	**95.6**	**94.7**–**95.8**	**2.5**	**2.0**–**2.8**	**1.9**	**1.5**–**2.2**
							
30–44	1432	93.4	92.0–94.6	2.9	1.9–3.6	3.7	2.7–4.7
45–59	1277	94.2	93.2–95.7	2.7	1.8–3.5	2.5	1.6–3.3
60–69	276	95.9	92.5–97.5	2.7	1.0–4.8	1.4	0.4–3.3
> 70	117	95.7	90.1–98.2	4.3	1.8–9.9	0	-
**Total (MEN)**	**3101**	**94.3**	**93.2**–**94.9**	**2.8**	**2.1**–**3.2**	**2.9**	**2.2**–**3.4**
							
30–44	1271	96.7	95.4–97.4	1.9	1.3–2.8	1.4	0.8–2.0
45–59	895	97.2	96.2–98.3	1.9	1.0–2.8	0.5	0.2–1.2
60–69	214	95.8	91.5–97.5	4.2	1.9–7.3	0	-
> 70	146	97.3	92.0–98.6	2.7	0.7–6.2	0	-
**Total (WOMEN)**	**2526**	**96.9**	**95.9**–**97.3**	**2.2**	**1.5**–**2.6**	**0.9**	**0.5**–**1.2**

bbreviations: CI (confidence interval). Percentages and CI’s are unadjusted.

**Table 5.** Transport-related activity among urban slum residents (SCALE-UP study).

Age group (years)	*N*	%	95% CI
			
30–44	2702	97.3	96.6–97.8
45–59	2172	96.7	95.8–97.4
60–69	490	92.8	90.2–94.8
> 70	263	84.4	79.4–88.3
**Total (ALL)**	**5627**	**96.0**	**95.5**–**96.5**
			
30–44	1432	98.5	97.8–99.0
45–59	1277	98.4	97.6–99.0
60–69	276	96	92.9–97.8
> 70	117	94	87.9–97.1
**Total (MEN)**	**3101**	**98.1**	**97.6**–**98.5**
			
30–44	1271	95.9	94.7–96.9
45–59	895	94.2	92.4–95.5
60–69	214	88.7	83.7–92.3
> 70	146	76.6	68.9–82.8
**Total (WOMEN)**	**2526**	**93.5**	**92.5**–**94.4**

Abbreviations: CI (confidence interval). Percentages and CI’s are unadjusted.

Transport-related physical activity was high among slum residents with 96.0% of respondents (98.1% men, and 93.5% women) indicating that they were spending most days in a typical week getting from place to place using active transportation (walking or cycling). Transport-related physical activity decreased when moving from younger to older age groups (Table 5).

**Figure 1.**
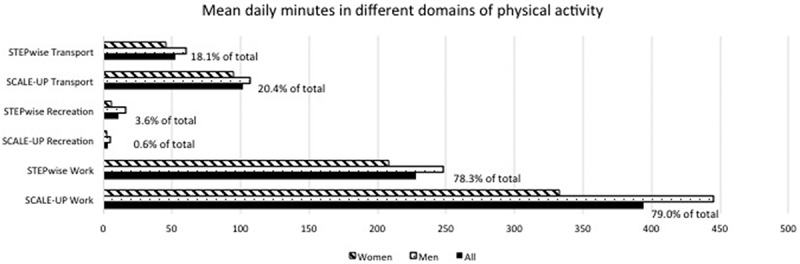
Mean daily minutes in different domains of physical activity for men (dotted) and women (striped), and all (black) in slum (SCALE-UP) and national (STEPwise) samples. The percentages reflect the proportion of the total time spent on activity per day.

In our comparison of different domains of self-reported physical activity, we found that the slum sample (SCALE-UP study) attained more mean daily minutes of total activity (499 minutes) compared to the national sample (291 minutes) (Figure 1). However, on investigation of the contributing proportions to total activity, both samples spent a similar proportion on work-related activities (79.0% for the slum sample [394 mean minutes for all, 445 for men, 333 for women] and 78.3% for the national sample [228 mean minutes for all, 248 for men, 208 for women]). Both samples also spent similar proportions of total activity on recreation-related activities (0.6% for the slum sample [3 mean minutes for all, 5 for men, 2 for women] and 3.6% for the national sample [11 mean minutes for all, 16 for men, 6 for women]), and on transport-related activities (20.4% for the slum sample [102 mean minutes for all, 107 for men, 95 for women] and 18.1% for the national sample [53 mean minutes for all, 60 minutes for men, 45 minutes for women]).

## Discussion

The analyses undertaken investigated several domains of self-reported activity among slum dwellers, for comparison to national activity level estimates. We found that the majority of slum dwellers reported spending a significant proportion of time on moderate and high levels of work-related activity, with only 7.1% reporting low levels of work-related physical activity. We also found that a large majority of slum dwellers (95.9%) had low levels of recreation-related physical activity, and that a very small proportion (4.0%) had low levels of transport-related physical activity. Compared to the national sample, slum residents reported 208 more daily mean minutes of total activity; however, both samples spent similar proportions of total activity on work-related activities (79.0% slum, 78.3% national), transport-related activities (20.4% slum, 18.1% national), and recreation-related activities (0.6% slum, 3.6% national). In both studies, the same questions were asked and the same examples regarding moderate and vigorous activity were included in the questions. While there maybe some differences in the interpretation of the questions or an over-reporting of the amount of time spent on the different activities by participants (which we would be unable to confirm), we think that this may indeed be a true reflection of the differences between slum residents and the national sample. The finding that both samples were spending the same proportion of time on the different activities lends further credence to this finding. Anecdotally, we know that slum residents spend hours walking to/from their places of work, often preferring to save their minimal pay for other more essential necessities like food and shelter. Many are also engaged in the informal sector, which requires large amounts of moderate-to-vigorous work-related activity. Our findings regarding physical activity in the slum setting are consistent with earlier research, in which most physical activity happened in the transport-related domain; recreation-related activity was reported less commonly [11–13]. However, contrary to Adlakha *et al*. [13] we did not find the opposite to be true for the national population. This difference could be explained because Adlakha *et al*. [13] compared physical activity levels between low- and high socio-economic-status neighborhoods, whereas we compared levels between slums and the national population.

Earlier research investigating patterns of physical activity and sedentary behavior in urban slum settings, found that involvement in physical activities decreased with age, higher sitting time was associated with physical activity, and women reported more on insufficient physical activity [11,12]. However, to the best of our knowledge, this is the first study comparing different domains of physical activity in a sample of slum residents to that of nationally representative findings. Given the significant differences in health experienced by slum dwellers compared to their non-slum urban or rural counterparts [8], for example weighing more and exercising less than rural dwellers but exercising more and being less obese than non-slum urban controls [14], it is imperative that NCD risk factors, such as physical inactivity, are assessed specifically in slum settings, rather than grouping them in with urban estimates.

### Limitations

The analyses relied on self-reported activity levels, however, objective measures would be more reliable. Self-report biases may have been diminished by providing examples of types of moderate and high intensity physical activities. In addition, there were sex, age, and education attainment distribution differences between the two studies which may influence the interpretation of findings. Nonetheless, this paper provides informative findings on domains of physical activity among slum dwellers, in comparison to national estimates.

### Perspectives for future planning

Whilst most slum dwellers already spend a great deal of time on transport-related physical activity, it is important that access to footpaths, with adequate street signage and lighting, is improved or maintained in such settings to encourage sustained active transportation through enhanced safety and street walkability [9]. This is also strongly recommended by UN-Habitat on the basis of observational studies [9,15]. Until now, this has been poorly studied in slum contexts, so further research is strongly needed on this topic [9]. Strategies to increase recreational physical-related activity could also be implemented, since only a small proportion of residents participate in this domain of activity. Studies have found that unsafe neighborhood conditions, lack of parks, playgrounds and open spaces, and financial barriers to access to recreational facilities (e.g. gyms, sports clubs, etc.) limit the ability of low socio-economic status populations to engage in leisure and recreational activities [8,10,16,17]. Increasing levels of recreational physical-related activity may in turn help to reduce stress levels (yet another NCD risk factor) and increase community cohesion, and may be achieved by measures such as providing safe spaces or fields for sporting activities that are accessible to all residents. For the elderly, this could also entail providing them with guidance and simple tools they may use to appropriately exercise at home in order to improve their health status. To the best of our knowledge, the latter has not yet been investigated.

### Conclusion

Taken together, our findings show that a lower proportion of urban slum residents reported low levels of physical activity compared to urban or national percentages, that slum dwellers accumulated significantly higher daily mean minutes of total activity, but that both slum and national samples spent similar proportions of total activity on work-, transport-, and recreation-related activities.

## Paper context

In Kenya, a nationally representative STEPwise survey of risk factors for non-communicable diseases established that 10.8% of Kenyans accumulated low levels of total physical activity. In a comparison of self-reported physical activity in two Nairobi slums to national estimates, the total amount of time spent, differs between urban slum residents and the national Kenyan population, although the proportions of time are similar. Such outcomes should be considered when addressing NCD risk factors in these populations.
